# The cerebellar-related cognitive function is sensitive to aging: implications for early detection

**DOI:** 10.3389/fnagi.2025.1679443

**Published:** 2025-12-09

**Authors:** Qianying Ma, Min Pu, Meijia Li, Ling Liu, Ruilin Wu

**Affiliations:** 1 Cognitive Science and Allied Health School, Beijing Language and Culture University, Beijing, China; 2 Key Laboratory of Language and Cognitive Science (Ministry of Education), Beijing Language and Culture University, Beijing, China; 3 Psychology Department, Vrije Universiteit Brussel and Center for Neuroscience, Brussels, Belgium; 4 Department of Decision Neuroscience and Nutrition, German Institute of Human Nutrition Potsdam-Rehbruecke, Potsdam, Germany; 5 Centre for Human Brain Health, School of Psychology, University of Birmingham, Birmingham, United Kingdom; 6 School of Humanities and Social Sciences, Beihang University, Beijing, China

**Keywords:** cerebellar cognitive affective syndrome (CCAS) scale, cerebellum, perceptual serial reaction time task (SRT task), aging, middle-aged adults, older

## Abstract

**Background:**

Accumulating evidence demonstrated that the cerebellum contributes to a wide range of sensorimotor and cognitive functions. However, the relationship between cerebellar function and cognitive abilities in normal aging populations remains unclear.

**Methods:**

The present cross-sectional study tested cerebellar-related cognitive changes across middle to late adulthood using the Cerebellar Cognitive Affective Syndrome (CCAS) scale and a perceptual serial reaction time (SRT) task. Participants were divided into three groups: early-middle-aged adults (*N* = 18, 30–45 years), late-middle-aged adults (*N* = 19, 46–57 years), and older adults (*N* = 18, 60–78 years).

**Results:**

Although all participants were identified as cognitively healthy by the Mini-Mental State Examination (MMSE), older adults showed significant impairments in the CCAS scale, especially in semantic and phonemic fluency, category switching, digit span backward as well as cube drawing/copy. In the perceptual SRT task, older adults responded slower than their counterparts, reflecting age-related impairments in sensorimotor integration efficiency. However, there were no age-related group differences in learning new procedural knowledge. Importantly, participants with poorer CCAS performance demonstrated slower response speed and lower accuracy in the perceptual SRT task.

**Discussion:**

The current results indicate a dissociation between general cognitive scores screened by MMSE and cerebellar-specific cognitive impairments. Furthermore, as the cerebellum plays a critical role in both sensorimotor and cognitive domains, the current study highlight the importance of incorporating screening tools which are sensitive to cerebellar functions in aging research.

## 1 Introduction

Given the rapidly increasing aging population across the world, understanding the aging brain and its subsequent impacts on cognitive functions is crucial for successful aging. While previous aging-related research has focused on the cerebral cortex, more and more recent research have demonstrated that the cerebellum also plays a vital role in the aging process (Arleo et al., 2024; Bernard et al., 2023).

Recent neuroimaging and neurophysiology studies have shown that the cerebellum contributes not only to the fine-tuning of voluntary movements but also to higher-order cognitive functions (Van Overwalle, 2024). Research have shown that healthy older adults have significantly reduced cerebellar gray matter volume compared to younger individuals (Bernard and Seidler, 2014), and such structural reductions correlate with decline of cognitive performance in aging (Uquillas et al., 2024). Moreover, functional magnetic resonance imaging (fMRI) studies have shown that lower cerebellar activation is linked to worse cognitive performance in tasks involving prediction and learning (Filip et al., 2019; Jackson et al., 2020). Furthermore, cerebellar atrophy and abnormal activity is evident even in the early stages of dementia and other neurodegenerative diseases (Chen et al., 2024; Gellersen et al., 2017; Tang et al., 2021). Also, the cerebellum may also be one of the brain structures that aging the fastest, with significant neuronal loss (McElroy et al., 2024). Overall, these findings suggest that the cerebellum may serve as a potential biomarker for early detecting aging-related decline and diagnosis of neurodegenerative diseases.

The Cerebellar Cognitive and Affective Syndrome (CCAS) scale is specifically developed to detect cognitive impairments associated with cerebellar dysfunction revealed by various neuroimaging studies, including linguistic processing, executive function, spatial cognition and affect regulation (Hoche et al., 2018). It uses different tasks to test related yet different cognitive functions as the Mini-Mental State Examination (MMSE), a standard screening tool used to evaluate global cognitive abilities (Li et al., 2016). For example, in the language domain, the MMSE includes naming common objects to test semantic knowledge/memory, which is mainly subserved by the cortical medial-temporal lobe (Saccuman et al., 2006). In the CCAS scale, language functioning is tested by semantic and phonemic fluency tasks. This executive retrieval of semantic knowledge is associated with the prefrontal cerebrocerebellar circuit (Hoche et al., 2018). Similarly, in the visuospatial domain, the MMSE uses a two-dimensional intersecting pentagon copying task, which requires minimal demand on spatial transformation abilities. In contrast, the CCAS scale includes a three-dimensional cube drawing/copying item, which relies more on cerebellar function related to spatial transformation and mental rotation (Cengiz and Boran, 2016). These indicate CCAS and MMSE may reveal different patterns in cognitive decline. However, research has primarily applied the CCAS to identify cerebellar impairment in clinical groups, leaving its relevance to healthy aging underdiagnosed.

In addition to the cognitive functions mentioned above, sequence learning is a fundamental cognitive ability that has been linked to cerebellar functions (Baetens et al., 2020; Hardwick et al., 2013; Ma et al., 2021). Research has demonstrated that sequence learning underlies various daily activities from basic acquisition of motor skills to higher-order function including language and social skills (Ma et al., 2021; Weiermann and Meier, 2012). Implicit sequence learning has received substantial interest in aging research, as impairments in this domain are commonly observed in people with neurodegenerative disorders, such as Parkinson’s disease (Firouzi et al., 2021; Gamble et al., 2014). Meanwhile, Bo et al. (2011) demonstrate that compared to younger participants, older adults have reduced cerebellar activation during motor sequence learning, while the observed cerebellar activations were associated with their learning performance. Furthermore, studies have shown that cerebellar stimulation using repetitive transcranial magnetic stimulation or anodal transcranial direct-current stimulation could enhance and consolidate sequence learning for healthy older adults (Khanmohammadi et al., 2025; Samaei et al., 2017). Overall, these findings indicate the close link between cerebellar function and sequencing learning. While prior research has emphasized motor sequence learning (e.g., sequential finger movements), perceptual sequence learning remains comparatively unexplored (Bo et al., 2011). Since impairments in motor learning may reflect either motor or learning deficits, examining perceptual sequence learning could bring more insights into understanding age-related changes in sequence acquisition and its underlying neural mechanisms.

Moreover, in aging studies, researchers quite often recruit extremely young adults (e.g., 18–25 years old) as a baseline to capture cognitive changes for older adults. However, this tends to overlook the critical period of middle adulthood, during which many age-related cognitive impairments begin to emerge. Recruiting middle-aged adults into aging studies may benefit from identifying preclinical markers of cognitive decline in later life. Therefore, the present study aimed to evaluate cerebellar-related cognitive functions using the CCAS scale and a perceptual sequence learning task for healthy individuals spanning early-middle to late adulthood. Overall, based on previous literature, we hypothesized that older adults would have lower CCAS scale scores and worse sequence learning performance compared to their younger counterparts, indicating their age differences in cerebellar-related functioning.

## 2 Methods

### 2.1 Study design and participants

*A priori* power analysis using G*Power (3.1.9.7; Faul et al., 2009) suggested that 54 participants were needed to achieve adequate power (1-*β* = 90%, *α* = 0.05) and a medium effect size *f* = 0.25 (i.e., η^2^ = 0.06) in this cross-sectional study. To account for potential attrition and exclusions due to cognitive impairment, a total of 58 candidates were recruited through online and local community advertisements in Beijing, Ningbo and Zhangjiakou between July 2024 and February 2025. This approach was intended to minimize selection bias and capture a more heterogeneous and representative sample than single-site recruitment.

Three participants were excluded due to low average accuracy (< 80%) across the perceptual serial reaction time task, resulting in a final sample of 55 participants. No participants were excluded based on MMSE screening scores (threshold ≤ 23 out of 30).^1^ In the following analyses, participants were grouped into three age groups: older (*n* = 18; 8 males; *M _age_* = 64.78, *SD* = 4.56, [60–78]), late-middle-aged (*n* = 19; 6 males; *M _age_* = 53.11, *SD* = 3.33, [46–57]), and early-middle-aged groups (*n* = 18; 3 males; *M _age_* = 36.83, *SD* = 5.48, [30–45]). All participants had normal or corrected-to-normal vision and no history of neurological or psychological disorders. Participants’ sex, education level, health condition and frequency of physical activities were recorded through online survey, and further confirmed by the experimenters at the beginning of the experiment (see Supplementary Table S1 for detailed demographic information). This study followed The Strengthening the Reporting of Observational Studies (STROBE) reporting guideline (von Elm et al., 2014, see Supplementary Appendix).

### 2.2 Experimental materials

Participants completed the MMSE and CCAS in a randomized order, followed by the perceptual SRT task. Between tasks, participants were allowed to rest at their own pace until they felt ready to proceed (See Supplementary Table S2 for main outcome summary). All experimenters involved in data collection received training to ensure consistent administration and scoring procedures.

#### 2.2.1 The mini-mental state examination

A general cognitive assessment was conducted using the Chinse version of the MMSE (Li et al., 2016), which includes domains of orientation, registration, attention and calculation, recall, as well as language and praxis.

#### 2.2.2 Cerebellar cognitive affective syndrome scale (CCAS)

In order to test cognitive and affective abilities related to the cerebellar functions, all participants completed the Chinese version of the CCAS scale (Guo et al., 2024; Liu et al., 2023). The CCAS has 10 items: semantic fluency, phonemic fluency, category switching, forward digit span, backward digit span, cube drawing/copy, verbal recall, similarities, Go/No-go, and affect. Each item can either be passed or failed. Cerebellar cognitive affective syndrome presence is classified as possible (one item failed), probable (two items failed), or definite (three items failed).

The Chinese version of the CCAS showed acceptable internal consistency in this study, with a Cronbach’s alpha of 0.70. This value is comparable to that reported in the original Chinese validation study including both cerebellar patients and healthy controls (Cronbach’s *α* = 0.72; Liu et al., 2023) and a large-scale Italian study in healthy individuals (Cronbach’s α = 0.70; Devita et al., 2025). These results support the use of this scale for efficiently assessing cerebellar-related cognitive functions.

#### 2.2.3 Perceptual serial reaction time task (perceptual SRT task)

**Figure 1:**
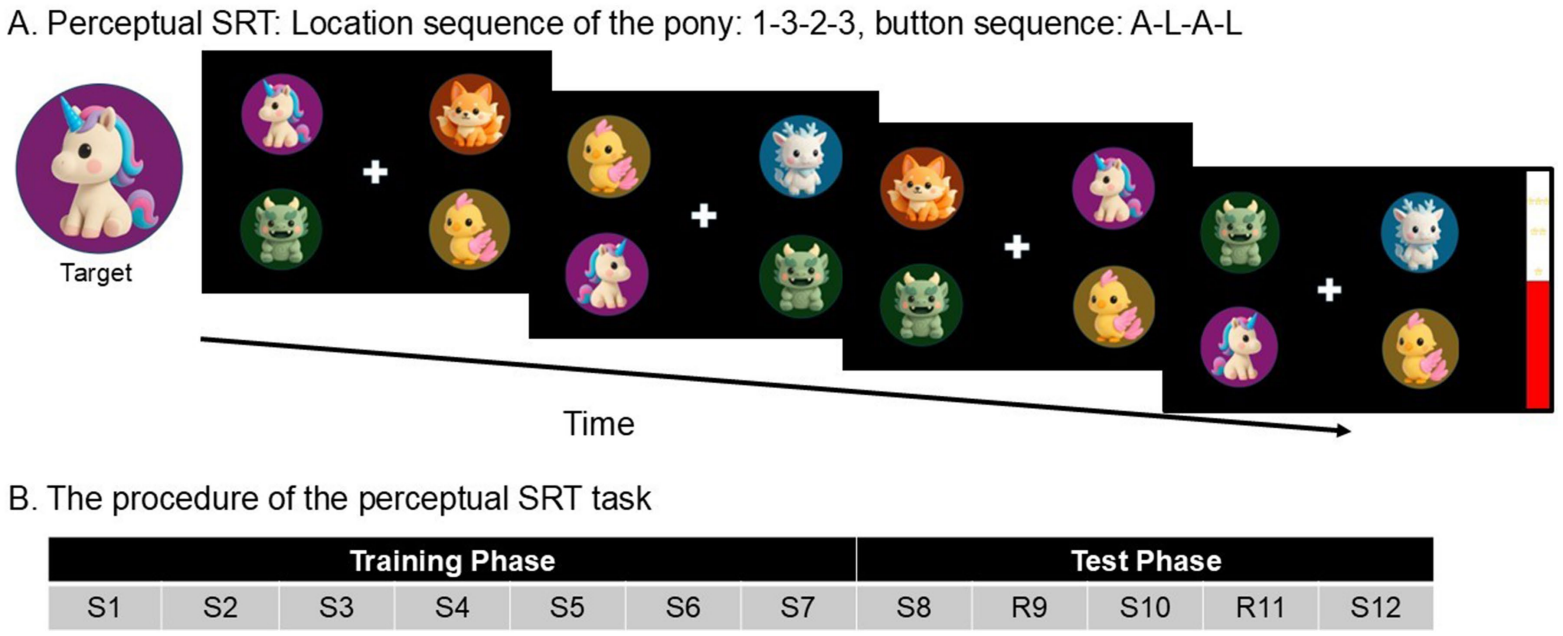
**(A)** A schematic example of the perceptual SRT task. On each trial, participants had to report the direction of the unicorn’s facing direction as quickly and correctly as possible by pressing “A” (left) and “L” (right). In the example of four consecutive trials, participants should press A-L-A-L. Unbeknownst to the participants, the unicorn’s location followed a repeating fixed sequence (1-3-2-3-4-2). The direction of the unicorn was completely random, making the response unpredictable from trial to trial, and dissociating perceptual sequence learning from motor responses. **(B)** The procedure of the perceptual SRT task. S, Standard block (=with a fixed sequence of target locations). R, Random block. Every block has a length of 60 trials.

In the perceptual SRT task, four cartoon animals appeared in screen quadrant (Figure 1A). Participants were instructed to respond “Is the unicorn facing left or right” as fast and as accurately as possible using keys “A” (left), and “L” (right) with their left and right index fingers. While the unicorn’s location on the screen followed a fixed sequence, its facing direction varied randomly, dissociating perceptual sequence learning from motor responses.

Each trial started after a 1,000 ms response–stimulus interval. When participants made errors or when they did not respond within 3,000 ms, a symbol of error “X” displayed for 750 ms on the screen. Participants received a break of 3,000 ms after each block.

A practice block of 12 trials preceded the main experiment, using a sequence that was different from the main experiment. Afterwards, the participants completed the Training phase (Figure 1B), consisting of Standard blocks 1–7 of 60 trials each (S1, S2, S3, S4, S5, S6, S7). Unbeknownst to participants, the locations of the unicorn in the perceptual SRT tasks appeared in a fixed 6-trial sequence (1–3–2-3-4-2), repeating 10 times per block. In the Training phase, the Standard block was repeatedly presented and so allowed implicit knowledge of the fixed sequence to develop. This was followed by a Test phase, during which the Standard block was occasionally interrupted by a Random block. The Test phase consisted of five blocks of 60 trials each (S8, R9, S10, R11, S12).

Participants’ learning performance was measured by: (1) General learning effect, participants would show a tendency to respond faster and faster (RT reduction) when the fixed sequence was repeated over the Training phase (i.e., S1–S7) even though they were never explicitly requested to learn anything; (2) Sequence-specific learning, participants would show slower reaction times (RT increase) when the fixed sequence was interrupted by a random sequence (i.e., R9 & R11), along with faster reaction time when the fixed sequence was presented again (S8, S10 & S12).

In order to assess participants’ explicit awareness of the fixed sequence, participants took a free recall test. They were asked to report whether they noticed something unusual during the experiment. Then, they were told that there was a fixed sequence, and they were encouraged to generate a 6-trial sequence of the unicorn’s location.

### 2.3 Statistical analysis

As MMSE and CCAS scales’ scores were not normally distributed (all Shapiro–wilk *ps* < 0.001), group differences were tested using Kruskal–Wallis H test, and significant effects were further tested by *post-hoc* comparisons with Bonferroni corrections. The effect size was calculated as *η^2^* = (H-k + 1)/N-K (*H*: Kruskal–Wallis test statistic; *k*: number of groups; *N*: total sample size; see also https://www.psychometrica.de/effektstaerke.html).

In the perceptual SRT tasks, when analyzing RTs, responses during and immediately after an error were excluded, as were trials with RTs shorter than 100 ms. Participants’ RTs were normally distributed. The general leaning effect was analyzed using mixed ANOVA with the training phase (S1 vs. S7) as a within-participants factor and age group (older, late-middle-aged, early-middle-aged) as a between-participants factor. The sequence-specific learning effect was analyzed using mixed ANOVA with block type (Random: average of R9 and R11; Standard: average of S8, S10, & S12) as a within-participants factor and age group as a between-participants factor. Significant effects were further tested by *post-hoc* comparisons with Bonferroni correction.

For all statistical analyses, the significance level was set to 0.05 with two-tailed tests. All analyses were conducted using SPSS version 29.

### 2.4 Role of the funding source

The funder of the study had no role in the study design, data collection, analysis and interpretation, or writing of the report.

## 3 Results

### 3.1 MMSE and CCAS

**Figure 2:**
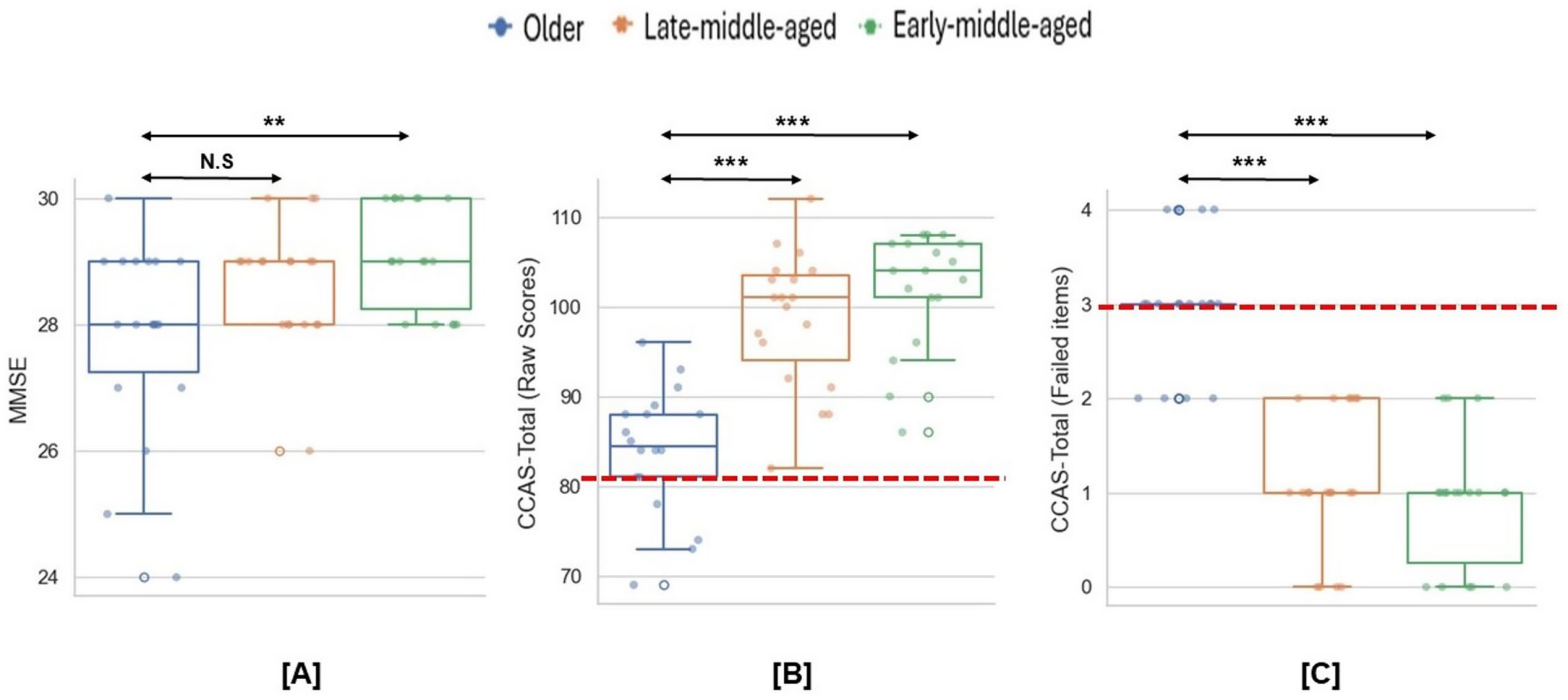
Group differences in the MMSE and CCAS scales. **(A)** Group differences in the MMSE scores. **(B,C)** Group differences in the CCAS raw scores and failed items. Dashed lines indicate the cut-off thresholds for the CCAS scale based on raw scores (< 82) and failed item counts (≥3). *p < 0.05; **p < 0.01; ***p < 0.001.

After the screening, 55 participants completed the experiment. As shown in Figure 2A, although no participants failed MMSE screening, older participants scored significantly lower than early-middle-aged adulthood [*χ^2^* = 8.64, *p* = 0.013, η^2^ = 0.13; *MD (Mean Difference)* early-middle-old = 1.23, *p* = 0.01, *Cohens’ d* = 1.04]. No age-related differences were found between other groups (all *ps* > 0.2).

As for the CCAS scale, the mean raw score was significantly lower for older than both younger groups (*χ^2^* = 29.54, *p* < 0.001, η^2^ = 0.53, *MD* early-middle-old = 18.06, *p* < 0.001, *Cohens’ d* = 2.66, *MD* late-middle-old = 14.63, *p* < 0.001, *Cohens’ d* = 1.99; Figure 2B). Meanwhile, compared to middle-aged participants, older participants had more items that scoring below the passing threshold (*χ^2^* = 34.57, *p* < 0.001, η^2^ = 0.63, *MD* early-middle-old = −2.11, *p* < 0.001, *Cohens’ d* = 3.08, *MD* late-middle-old = −1.90, *p* < 0.001, *Cohens’ d* = 2.64; Figure 2C). In total, 14 older participants (78%) meet the criteria for a definite CCAS (i.e., ≥3 failed items). Specifically, older participants performed significantly worse on semantic and phonemic fluency, category switching, digital span backward and cube drawing/copy (Table 1).

**Table 1:** Group differences in cerebellar cognitive affective syndrome scale.

Items	Older (*N* = 18)		Late-middle-aged (*N* = 19)		Early-middle-aged (*N* = 18)		Pass/Max scores
	Mean score	Number of participants failed test	Mean score	Number of participants failed test	Mean score	Number of participants failed test	
Language processing test							
Semantic fluency	19.67 ± 3.87	3 (16.67%)	23.32 ± 2.91	0 (0%)	24.50 ± 2.83	0 (0%)	16/26
	*χ^2^* = 15.72, *p* < 0.001, η^2^ = 0.26, *MD early-middle-old* = 4.83, *p* < 0.001, *Cohens’ d* = 1.43; *MD late-middle-old* = 3.65, *p* = 0.019, *Cohens’ d* = 1.07						
Phonemic fluency	6.78 ± 1.70	18 (100%)	9.11 ± 1.79	11 (57.89%)	8.67 ± 1.75	10 (55.56%)	10/19
	*χ^2^* = 13.67, *p* = 0.001, η^2^ = 0.22 *MD early-middle-old* = 1.89, *p* = 0.016, *Cohens’ d* = 1.10; *MD late-middle-old* = 2.33, *p* = 0.001, *Cohens’ d* = 1.33						
Executive function test							
Mental flexibility							
Category switching	9.72 ± 2.24	11 (61.11%)	11.89 ± 2.40	3 (15.79%)	13.61 ± 2.23	2 (11.11%)	10/15
	*χ^2^* = 18.64, *p* < 0.001, η^2^ = 0.32 *MD early-middle-old* = 3.89, *p* < 0.001, *Cohens’ d* = 1.74; *MD late-middle-old* = 2.17, *p* = 0.034, *Cohens’ d* = 0.93						
Working memory							
Digital span forward	7.61 ± 0.61	0 (0%)	7.84 ± 0.38	0 (0%)	7.94 ± 0.24	0 (0%)	6/8
	*χ^2^* = 4.87, *p* = 0.088, η^2^ = 0.06						
Digital span backward	4.28 ± 1.13	5 (27.78)	5.26 ± 0.73	0 (0%)	5.11 ± 0.90	0 (0%)	4/6
	*χ^2^* = 8.39, *p* = 0.015, η^2^ = 0.12 *MD late-middle-old* = 0.99, *p* = 0.019, *Cohens’ d* = 1.04						
Verbal recall	10.89 ± 2.81	6 (33.33%)	12.53 ± 1.71	1 (5.26%)	12.56 ± 1.42	0 (0%)	11/15
	*χ^2^* = 4.83, *p* = 0.089, η^2^ = 0.05						
Inhibition							
Go/No-go	1.61 ± 0.78	3 (16.67%)	1.89 ± 0.32	0 (0%)	2 ± 0	0 (0%)	1/2
	*χ^2^* = 4.75, *p* = 0.093, η^2^ = 0.05						
Abstract reasoning							
Similarities	6.06 ± 1.96	8 (44.44%)	7.21 ± 0.98	4 (21.05%)	7.17 ± 0.79	4 (22.22%)	7/8
	*χ2* = 3.56, *p* = 0.17, η^2^ = 0.03						
Visual–spatial ability test							
Cube drawing/copy	11.39 ± 2.87	3 (16.67%)	13.58 ± 2.12	1 (5.26%)	14.50 ± 1.15	0 (0%)	12/15
	*χ^2^* = 20.06, *p* < 0.001, η^2^ = 0.35 *MD early-middle-old* = 3.11, *p* < 0.001, *Cohens’ d* = 1.42; *MD late-middle-old* = 2.19, *p* = 0.007, *Cohens’ d* = 0.87						
Affect regulation test							
Affective	6 ± 0	0 (0%)	6 ± 0	0 (0%)	6 ± 0	0 (0%)	5/6
	--						

### 3.2 Explicit awareness of the perceptual SRT task

Sequence awareness was assessed post-experiment. Only one early-middle-aged participant reported noticing something unusual without pointing out the sequence. No participants successfully reproduced the full sequence. Three participants in the older, five in the late-middle-aged and one in the early-middle-aged group successfully reproduced sequence segments. Average correct sequence recollections were 2 for the older group, 2.6 for the late-middle-aged group and 2 for the early-middle-aged group. Therefore, no participant was excluded for high explicit awareness.

### 3.3 General learning effect during training phase

**Figure 3:**
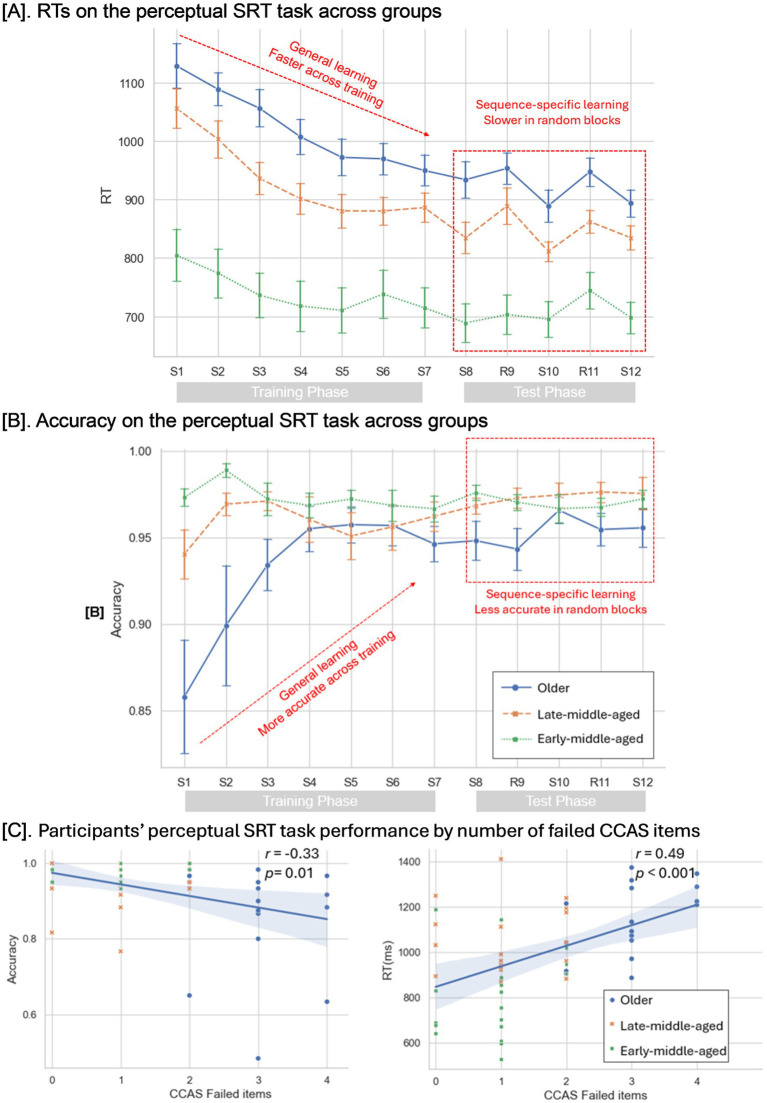
Performance on the perceptual SRT task. **(A)** The mean RTs on the perceptual SRT task across the experiment. **(B)** The mean accuracy rate on the perceptual SRT task across the experiment. Error bars = Standard errors of the mean. **(C)** The correlations between the beginning performance on perceptual SRT and the numbers of failed CCAS items.

For the RTs (Figure 3A), the mixed ANOVA analysis demonstrated a main effect of general learning (*F*_(1,52)_ = 81.33, *p* < 0.001, *η^2^* = 0.61), indicating progressive RT reduction across repetitive training blocks. A main effect of age group (*F*_(2,52)_ = 21.71, *p* < 0.001, *η^2^* = 0.46) was also found, indicating faster responses of the early-middle-aged adults compared to other two groups (*MD* Late-middle–Early-middle = 212 ms, *p* < 0.001, *Cohens’ d* = 6.85, 95%CI [103, 320]; *MD* Old-Early-middle = 280 ms, *p* < 0.001, *Cohens’ d* = 8.92, 95%CI [170, 389]). No interaction was found between the general learning and groups (*p* > 0.05).

For accuracy (Figure 3B), the analysis revealed increased accuracy across training (*F*_(2,52)_ = 6.84, *p* = 0.01, *η^2^* = 0.12). Meanwhile, older participants respond less accurately compared to the two groups (*F*_(2,52)_ = 10.13, *p* < 0.001, *η^2^* = 0.28; *MD* Late-Middle-Old = 4.9%, *p* = 0.007, *Cohens’ d* = 5, 95%CI [1.1, 8.7%]; *MD* Early-middle-Old = 6.8%, *p* < 0.001, *Cohens’ d* = 7, 95%CI [2.9, 10.6%]). There was a significant interaction between general learning and age group (*F*_(2,52)_ = 4.44, *p* = 0.02, *η^2^* = 0.15). Post-hoc tests showed that older participants showed significant accuracy improvement through training but not for the other two groups (*MD*_S7–S1_ = 8.8%, *p* < 0.001, *Cohens’ d* = 3.04, 95%CI [4.2, 13.5%]), demonstrating their preserved learning capacity despite slower initial performance.

### 3.4 Sequence-specific learning during test phase

For RTs (Figure 3A), mixed ANOVA analysis revealed a main effect of sequence-specific learning shown by slower responses to random sequences compared to the fixed ones (*F*_(1,52)_ = 41.64, *p* < 0.001, *η^2^* = 0.45). Meanwhile, the early-middle-aged group responded faster than both older groups (*F*_(2,52)_ = 19.39, *p* < 0.001, *η^2^* = 0.43; *MD* Late-Middle–Early-middle = 142 ms, *p* < 0.001, *Cohens’ d* = 5.68, 95%CI [55, 259]; *MD* Old-Early-middle = 219 ms, *p* < 0.001, *Cohens’ d* = 8.76, 95%CI [131, 307]). No significant interaction was observed (*p* > 0.1).

As shown in Figure 3B, the analysis showed that older participants responded less accurately than middle-aged participants (*F*_(2,52)_ = 5.08, *p* = 0.01, *η^2^* = 0.16, *MD* Late-Middle-Old = 2.1%, *p* = 0.01, *Cohens’ d* = 2.4, 95%CI [0.4%, 3.8]), without no main effect of learning or any interactions.

### 3.5 CCAS performance and SRT task

Participants were regrouped by the number of failed items on the CCAS scale (Figure 3C). Given the unequal group sizes (0 failures: *N* = 9; 1 failure: *N* = 19; 2 failures: *N* = 13; 3 failures: *N* = 14), no mixed-ANOVA tests were performed. The Spearman correlation analyses showed that participants with more CCAS failed items tended to have slower RTs and lower accuracy during the initial block (RTs: *r* = 0.49, *p* < 0.001, 95%CI [0.25, 0.67]; Accuracy: *r* = −0.33, *p* = 0.01, 95%CI [−0.55, −0.07]).

## 4 Discussion

In the current study, while all participants demonstrated normal MMSE performance, their CCAS scores revealed distinct patterns. Specifically, no significant difference was observed in MMSE scores between late-middle-aged participants and those aged above 60 years. In contrast, older participants showed worse performance on CCAS scale than the early-middle-aged and the late-middle-aged participants. Additionally, there was a marked age-related deficit in reaction times during the perceptual SRT task, and participants responded slower with more failed items in CCAS scale. Overall, these results suggest that cerebellar-mediated cognitive functions may be particularly vulnerable to aging process, with further potential screening sensitivity within older subgroups.

### 4.1 Cerebellar-related cognition during aging process

In the current study, older adults had significantly lower scores in the cerebellar-related cognitive screening than younger participants. This finding aligns with previous research, which has shown an increase in failed CCAS items and lower CCAS raw scores with age, particularly in adults over 60 (Schmahmann et al., 2021; Thieme et al., 2021). Also, consistent with earlier studies, we observed that adults who passed the MMSE could still fail on the CCAS scale (Devita et al., 2025; Schmahmann et al., 2021; Thieme et al., 2021). Therefore, this suggests the CCAS may capture early and subtle cerebellar-mediated deficits prior to global cognitive impairment in normal aging individuals (Alan et al., 2024; Devita et al., 2025; Hoche et al., 2018).

Also, consistent with prior reports, older participants showed significant impairments in digital span backward and cube drawing/copy (Hoche et al., 2018; Thieme et al., 2021). This reflected cerebellar contributions to search and transformation processes rather than simple information storage. Notably, older participants had clear deficits in both semantic and phonemic fluency, consistent with prior evidence that verbal fluency is sensitive to aging processing (Frankenberg et al., 2021). Moreover, the current study indicated that phonemic fluency was more impaired than semantic fluency in older adults. Previous studies suggest that phonemic tasks have high demands on executive functions due to sustained attention to consonants, applying and matching phonemic rules, and inhibiting semantic associations (Molinari and Leggio, 2016; Silveri, 2021). Clinical evidence further indicates that phonemic fluency involves greater cerebellar engagement, as cerebellar patients had disproportional deficits in phonemic than semantic fluency (Molinari and Leggio, 2016; Peterburs et al., 2010; Silveri, 2021). This may explain why even the early-middle-aged participants in the current study started to show low performance on this subscale.

However, 56% of the early-middle-aged participants who had college degrees did not meet the passing threshold for phonemic fluency, this finding indicates that linguistic background also influences participants’ performance. In previous validation study of Chinese CCAS scale, Chinese cerebellar patients had average scores of 3.30 in phonemic fluency (Guo et al., 2024), which is evidently lower than scores reported in the original English-speaking CCAS cohort (Hoche et al., 2018). Other studies based on Chinese populations showed young adults (age around 20) produce approximately 13 words (Sun et al., 2022) whereas healthy older individuals (age around 66) produce 5 or 6 words in phonemic tasks (Li et al., 2021). These findings indicate a more lenient threshold should be adapted according to mandarin context in the phonemic fluency subscale, which could help reduce the potential false positive rate. Overall, our results further highlight the need for large-scale normative studies of the CCAS scale across the lifespan in the Chinese population to establish appropriate age-stratified cutoffs.

Note that excluding the phonemic fluency subscale did not alter the main findings of the current study. Older participants still present more failed items than middle-aged groups, and those with more CCAS failed items have slower RTs and lower accuracy (See Supplementary material). Regardless of the threshold used for this subscale, age differences among the three groups were significant in the number of words generated per minute, indicating that verbal fluency, especially phonemic fluency, is sensitive to aging process.

It is also noteworthy that no age-related effects were observed on the Go/No-go or verbal recall items in the current sample. Clinical studies have identified memory impairments in populations with cerebellar damages such as stroke or ataxia or Chiari malformation using comparable tests (Allen et al., 2018; Hoche et al., 2018; Van Overwalle et al., 2019). The absence of age effects could be due to those items are relatively easy in CCAS scale. Another explanation is that deficits in inhibition and memory associated with cerebellar pathology may appear more pronounced than those seen in normal aging. This suggests that clinically significant impairments in these domains may serve as indicators of underlying cerebellar pathology dysfunction.

The current study also evaluated perceptual sequence learning ability from middle to late adulthood. While some previous studies reported impaired motor sequence learning abilities in aging population, others found no such deficits (Bo et al., 2011; Weiermann and Meier, 2012). However, these studies combined motor execution and sequence learning, as motor responses were confounded with the sequences. By using the perceptual SRT task, the current study could dissociate the motor and non-motor learning processes. Our results showed age-related declines in general sensorimotor integration efficiency (i.e., slower button-pressing speeds), and perceptual learning ability remained largely intact in older adults. This dissociation may reflect a selective decline of neural plasticity. While learning associated with rapid response and new memory formation may be sensitive to aging process, the plasticity underlying perceptual skills in subcortical regions may not have significant age-related group differences. This also highlight future research on mechanisms of selective neural preservation and cerebellar reserve (Li et al., 2023; Mitoma et al., 2020).

Additionally, in line with previous studies showing a link between poor learning and reduced cerebellar volume or activation (Bo et al., 2011; Hicks et al., 2025), participants with more CCAS failures demonstrated slower reaction times and lower accuracy. Descriptively, older participants required four blocks to achieve accuracy levels that younger participants reached in the first block. This pattern suggests that the normal aging cerebellum may support individuals to maintain their capacity for procedural learning but operate with reduced efficiency (Filip et al., 2019; Hicks et al., 2025). This slower but ultimately successful learning pattern suggests that given adequate time and practice, aging populations can acquire new procedural knowledge. This further supports theories of maintained neuroplasticity and the cerebellar reserve function in later life (Bernard, 2022; Mitoma et al., 2020). Thus, combining SRT tasks and neuroimaging may provide deeper insights into the role of the cerebellum in learning abilities in aging populations. At the practical level, these results imply that successful aging interventions should include opportunities for novel skill acquisition, rather than focusing solely on the rehearsal of existing knowledge, in order to create cognitively enriched environments.

To gain a more comprehensive understanding of cerebellar-related cognitive domains throughout adulthood, we included middle-aged participants in the study. The CCAS scale revealed older people had worse performance compared to the late-middle-aged participants whereas the MMSE could not distinguish these two groups. This age-related group difference in cognitive performance was consistent with a recent large-scale neuroimaging study, which showed the first brain age-related change peaks at 57 years old (Liu et al., 2024). In the perceptual SRT task, late-middle-aged participants showed slower RTs than younger adults yet presented comparable accuracy levels. They also outperformed the older group in accuracy and speed. As mentioned above, processing speed is highly sensitive to aging. An alternative explanation is that individuals may prioritize accuracy over speed, a prudent strategic adaptation during aging that helps preserve performance (Vallesi et al., 2021). At the neuropsychological level, the relatively intact cerebellar function observed in late-middle-aged participants, as indicated by their CCAS scores, may help compensate for incipient age-related difficulties in completing SRT tasks. This interpretation aligns with neuroimaging evidence suggesting that cerebrocerebellar connectivity within functional networks predicts cognitive processing speed (Li et al., 2022), and that increased connectivity serves as a compensatory mechanism when the cerebellum recruiting additional neural resources to support cognitive processing. With more severe deficits during aging, compensation is not enough, and individuals are likely to show more CCAS failures alongside slower and less accurate task performance.

Moreover, both late-middle-aged and older adults presented greater variability in their scores compared to the younger group for screening scales and the perceptual SRT task. This suggests that while group-level functional differences may become evident around age 60 (Liu et al., 2024), inter-individual differences in the trajectory of decline may be detectable as early as midlife. This interindividual variability likely extends beyond normative age-related individual differences and may partially indicate subclinical cognitive changes, particularly in the absence of objective neuroimaging or biomarker data (Devita et al., 2025). Also, there is a need to focus and track on people who show abnormal cognitive impairments during their middle adulthood compared to their peers. By doing this, we could have an improved understanding of cerebellar function during middle adulthood, which may help to identify early markers of cerebellar decline and potential disease transitions.

### 4.2 Limitations and implication

This study has several limitations. First, although a power analysis indicated the sample size was adequate for statistical testing, the relatively small number of participants in each group may limit the generalizability of the findings, particularly given the high interindividual variability inherent in aging. Therefore, future large-scale multi-center studies are needed to further explore the relationship between aging and cerebellar-mediated cognitive functions.

Second, the relatively high selectivity of the CCAS may lead to false positive (Thieme et al., 2021), potentially misclassifying cognitively healthy individuals as impaired. We maintained the strict criteria as the original CCAS design (Hoche et al., 2018) and a large-scale screening in healthy populations (Devita et al., 2025). Therefore, it is unlikely to affect our findings, as only older adults showed definite CCAS difficulties, and their total and subscale scores were significantly lower than those of the younger groups. Nevertheless, our results emphasize that future studies should adapt the CCAS scale to account for cultural and language contexts, and establish normative age-stratified cutoffs.

Third, the insensitivity of the MMSE to mild cognitive impairment (MCI, Arevalo-Rodriguez et al., 2013) means that some participants in the current sample may have had preclinical deficits, limiting the generalization of our findings. External validity requires verification in more diverse populations. Further studies should recruit various groups including individuals with subjective cognitive decline, MCI and dementia in order to clarify how cerebellar functional decline contributes to both healthy and pathological aging.

Fourth, the study inferred cerebellar involvement based on behavioral measures of the CCAS. In the absence of structural or functional neuroimaging data, it remains uncertain to what extent variability in CCAS scores directly reflects cerebellar integrity. Therefore, the attribution of observed cognitive deficits specifically to cerebellar dysfunction, though theoretically linked, remains indirect. Future studies should incorporate multimodal neuroimaging, including structural and functional MRI, to objectively quantify anatomical and functional differences between individuals who pass the MMSE but fail the CCAS and those who pass both assessments. By doing this, future research could explore whether CCAS performance effectively serves as a behavioral indicator of cerebellar status in normal aging. This approach may also highlight a potential target area for neuro-intervention in age-related neurodegenerative diseases.

Fifth, the current study used free recall to measure participants’ explicit sequence awareness. Although participants showed limited awareness of sequence existence, this method may not be sensitive enough to capture participants’ implicit sequential knowledge. Future aging research with SRT tasks could apple a more structured post-test interview or a recognition task (Destrebecqz and Cleeremans, 2001).

## 5 Conclusion

In summary, the current study provides direct evidence of significant cerebellar-related cognitive decline during aging, potentially underlying common aging patterns, such as slower responses and poor executive language processing. Our findings suggest that successful aging depends as much on cerebellar health as on cortical integrity. Recognizing the cerebellum’s unique role in cognitive aging enables the development of more comprehensive assessment approaches and more targeted interventions for promoting healthy aging.

## Data Availability

All (pseudonymized or anonymous) data are available upon request from the corresponding author(s), excluding data that allow identifying individual participants.

## Footnotes

1 Different cutoff scores are used for MMSE. In this study, we used the cutoff recommended by the National Health Commission of the People’s Republic of China in the document *The standard for healthy Chinese older adults* (WS/T 802—2022).
