# Supplementary material for: The cerebellar-related cognitive function is sensitive to aging: implications for early detection
Source: Front Aging Neurosci. 2025 Dec 9;17:1679443. doi: 10.3389/fnagi.2025.1679443 (PMC12722794; doi:10.3389/fnagi.2025.1679443)
Supplement: Supplementary file 1 [file Data_Sheet_1.PDF]

The cerebellar-related cognitive function is sensitive to aging: Implications for early detection

Supplementary file

Qianying Ma[1,2,3], Min Pu[4], Meijia Li[5], Ling Liu[1,2], Ruilin Wu[6]

1. Cognitive Science and Allied Health School, Beijing Language and Culture University, Beijing, China
2. Key Laboratory of Language and Cognitive Science (Ministry of Education), Beijing Language and Culture University, Beijing, China
3. Psychology Department, Vrije Universiteit Brussel and Center for Neuroscience, Brussels, Belgium
4. Department of Decision Neuroscience and Nutrition, German Institute of Human Nutrition Potsdam-Rehbruecke, Potsdam, Germany
5. Centre for Human Brain Health, School of Psychology, University of Birmingham, Birmingham, UK
6. School of Humanities and Social Sciences, Beihang University, Beijing, China

Supplementary Table S1. Participant Demographic Information

	Older	Late-middle-aged	Early-middle-aged
Sample size	18	19	18
Age	64.78 ± 4.56, [60-78]	53.11 ± 3.33, [46-57]	36.83 ± 5.48, [30-45]
Gender (M/F)	8/10	6/13	3/15
Education level (Number of participants)	Elementary school: 6<br>High school: 11<br>University and above: 1	Elementary school: 4<br>High school: 9<br>University and above: 6	Elementary school: 0<br>High school: 0<br>University and above: 18
Self-rating health condition (Number of participants)	Fair: 7<br>Good: 8<br>Very Good: 3	Fair: 10<br>Good: 7<br>Very Good: 2	Fair: 5<br>Good: 11<br>Very Good: 2
Frequency of physical activities (Number of participants)	Everyday: 6<br>A few times per week: 4<br>Once per week: 2<br>One to three times per month: 0<br>Seldom: 5<br>Never: 1	Everyday: 6<br>A few times per week: 4<br>Once per week: 2<br>One to three times per month: 2<br>Seldom: 4<br>Never: 1	Everyday: 2<br>A few times per week: 5<br>Once per week: 1<br>One to three times per month: 2<br>Seldom: 4<br>Never: 4

Supplementary Table S2. Outcome summary

MMSE	Orientation; Registration; Attention and calculation; Recall; Language and praxis
CCAS	Semantic fluency; Phonemic fluency; Category switching; Forward digit span, Backward digit span; Cube drawing/copy; Verbal recall; Similarities; Go/No-go; Affect
Perceptual SRT	General learning: faster reaction times during the Training phase
	Sequence-specific learning: Slower reaction times in Random blocks compared to Sequenced blocks

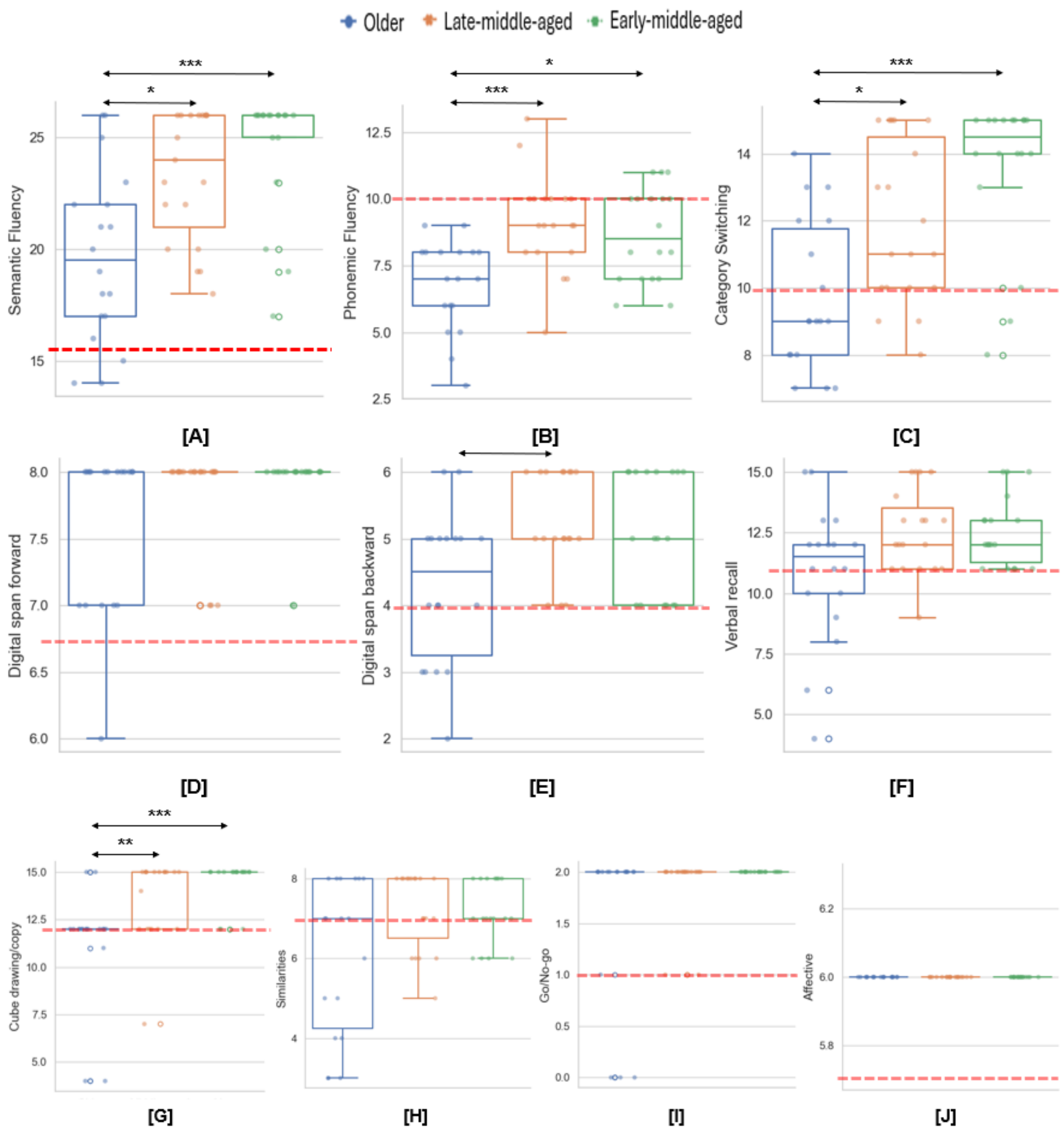

Figure S1. Group differences in each CCAS item. Grey dashed lines indicate the cut-off thresholds for the CCAS subscales. *: $p < 0.05$; **: $p < 0.01$; ***: $p < 0.001$.

Appendix:

STROBE Statement—Checklist of items that should be included in reports of ***cross-sectional studies***

	Item No	Recommendation
**Title and abstract**	1	(*a*) Indicate the study's design with a commonly used term in the title or the abstract
		Page 1
		(*b*) Provide in the abstract an informative and balanced summary of what was done and what was found
		Page 1
**Introduction**		
Background/rationale	2	Explain the scientific background and rationale for the investigation being reported
		Introduction, Paragraph 1-4
Objectives	3	State specific objectives, including any prespecified hypotheses
		Introduction, the last Paragraph
**Methods**		
Study design	4	Present key elements of study design early in the paper
		Method, Section 2.1, Paragraph 1
Setting	5	Describe the setting, locations, and relevant dates, including periods of recruitment, exposure, follow-up, and data collection
		Method, Section 2.1, Paragraph 1
Participants	6	(*a*) Give the eligibility criteria, and the sources and methods of selection of participants
		Method, Section 2.1
Variables	7	Clearly define all outcomes, exposures, predictors, potential confounders, and effect modifiers. Give diagnostic criteria, if applicable
		Method, Section 2.2
Data sources/ measurement	8*	For each variable of interest, give sources of data and details of methods of assessment (measurement). Describe comparability of assessment methods if there is more than one group
		Method, Section 2.2
Bias	9	Describe any efforts to address potential sources of bias

		Method, Section 2.1 & 2.2
Study size	10	Explain how the study size was arrived at
		Method, Section 2.1, Paragraph 1
Quantitative variables	11	Explain how quantitative variables were handled in the analyses. If applicable, describe which groupings were chosen and why
		Method, Section 2.1, Paragraph 2
Statistical methods	12	(*a*) Describe all statistical methods, including those used to control for confounding
		Method, Section 2.3
		(*b*) Describe any methods used to examine subgroups and interactions
		NA
		(*c*) Explain how missing data were addressed
		NA
		(*d*) If applicable, describe analytical methods taking account of sampling strategy
		Method, Section 2.3
		(*e*) Describe any sensitivity analyses
		Method, Section 2.3
**Results**		
Participants	13*	(a) Report numbers of individuals at each stage of study—eg numbers potentially eligible, examined for eligibility, confirmed eligible, included in the study, completing follow-up, and analysed
		Method, Paragraph 1-2; Result, Paragraph 1
		(b) Give reasons for non-participation at each stage
		NA
		(c) Consider use of a flow diagram
		NA
Descriptive data	14*	(a) Give characteristics of study participants (eg demographic, clinical, social) and information on exposures and potential confounders
		Supplementary Table S1
		(b) Indicate number of participants with missing data for each variable of interest

		No missing data
Outcome data	15*	Report numbers of outcome events or summary measures
		Results, Section 3.1 & 3.3, Figure 2-3, Table 1, and Supplementary Table S2
Main results	16	(*a*) Give unadjusted estimates and, if applicable, confounder-adjusted estimates and their precision (eg, 95% confidence interval). Make clear which confounders were adjusted for and why they were included
		Results, section 3.3-3.5
		(*b*) Report category boundaries when continuous variables were categorized
		Results, Table 1
		(*c*) If relevant, consider translating estimates of relative risk into absolute risk for a meaningful time period
		NA
Other analyses	17	Report other analyses done—eg analyses of subgroups and interactions, and sensitivity analyses
		Results, section 3.5, Figure 3C
**Discussion**		
Key results	18	Summarise key results with reference to study objectives
		Discussion, Paragraph 1
Limitations	19	Discuss limitations of the study, taking into account sources of potential bias or imprecision. Discuss both direction and magnitude of any potential bias
		Discussion, Section 4.2
Interpretation	20	Give a cautious overall interpretation of results considering objectives, limitations, multiplicity of analyses, results from similar studies, and other relevant evidence
		Discussion, Section 4.2
Generalisability	21	Discuss the generalisability (external validity) of the study results
		Discussion, Section 4.2; Conclusion
**Other information**		
Funding	22	Give the source of funding and the role of the funders for the present study and, if applicable, for the original study on which the present article is based
		Method, Section 2.4

*Give information separately for exposed and unexposed groups.

Note: An Explanation and Elaboration article discusses each checklist item and gives methodological background and published examples of transparent reporting. The STROBE checklist is best used in conjunction with this article (freely available on the Web sites of PLoS Medicine at http://www.plosmedicine.org/, Annals of Internal Medicine at http://www.annals.org/, and Epidemiology at http://www.epidem.com/). Information on the STROBE Initiative is available at www.strobe-statement.org.
